# Lived experiences matter: The role of mental health professionals’ psychological crises and vulnerability in shaping their health beliefs and concepts

**DOI:** 10.3389/fpsyt.2023.1114274

**Published:** 2023-01-25

**Authors:** Angel Ponew, Anna Brieger, Christian Lust, Sven Speerforck, Sebastian von Peter, Stefan Stuetzle

**Affiliations:** ^1^Department of Psychiatry and Psychotherapy, Medical University Brandenburg Theodor Fontane, Neuruppin, Germany; ^2^Department of Psychiatry and Psychotherapy, University of Leipzig Medical Center, Leipzig, Germany; ^3^Evangelische Hochschule Dresden, University of Applied Sciences for Social Work, Education and Care, Dresden, Germany

**Keywords:** mental health professionals, lived experience, vulnerability, causal beliefs, health concept, depression, burnout, stigmatization

## Abstract

**Background:**

Mental health professionals are often affected by mental health problems and disorders. Yet, the effects of these lived experiences on their causal beliefs and health concepts have not been investigated. The current study investigates how professionals’ lived depressive experiences and their perceived vulnerability to mental illness affect their causal beliefs about mental disorders, their general concept of mental health and their specific concepts of depression and burnout.

**Methods:**

An online survey was conducted with 218 mental health professionals from 18 psychiatric clinic departments in the German federal states of Berlin and Brandenburg, investigating their experiences with depression, self-assessed vulnerability, their causal beliefs of mental illness, their general health concept and specific illness concepts of depression and burnout. A path model was calculated to examine the relationships between these variables. Participants with and without lived experience of depression were grouped.

**Results:**

Lived experience of depression was indicated by 126 participants. For participants with no experience of depression, perceived vulnerability negatively predicted beliefs in biological causation, which positively predicted higher differentiation between depression and burnout. For participants with previous depression experiences, perceived vulnerability positively predicted beliefs in psychological and social causation. Continuum belief was predicted only in this group by the three variables of causal beliefs. Psychological and social causation was positively associated, while biological causes were negatively associated with continuum beliefs.

**Conclusion:**

Mental health professionals are not external to the clinical situation. Their lived experiences do matter, shaping their beliefs and concepts and, thus, possibly also their actions toward patients.

## 1. Introduction

Mental health care professionals’ attitudes toward psychiatric classificatory concepts and, more generally, toward the nature of mental health are shaped by their health concepts and beliefs. For instance, the belief in a biological or biogenetical causation of mental disorders (i.e., genetic or physiological factors like heritability, chemical changes, or brain abnormalities) may influence professionals’ attitudes in terms of treatability, prognosis, and treatment decisions (1, 2), or of empathy toward their patients (3). Similarly, the assumption that mental health and illness are either separated by a clear cutoff (categorical conceptualization) or both located within a continuum (continual conceptualization) can have an impact on mental health staff’s attitudes toward service users and their mental health issues (4, 5). For example, a categorial view of mental health is associated with more stigmatizing attitudes (6, 7), which are known to result in discriminatory behavior (8).

At the same time, it is unclear if and in what way the attitudes of mental health care professionals toward classificatory entities are also shaped by personal factors, like their experiences living with mental health problems or their self-perceived vulnerability to mental health problems. This lack of insight surprises even more as a slowly growing body of evidence demonstrates a substantial frequency of mental health problems and disorders among mental health professionals (9–11). In this context, a German study (EKB study) found that over 80% of a self-selected sample of mental health professionals stated to have experienced mental crisis including mental disorders (12). At the same time, this study showed that crises-experienced mental health professionals, despite sharing certain experiences with their patients, did not identify with these, but felt a strong need to disidentify from them.

These and other studies indicate that a substantial portion of mental health care professionals reports lived experiences from the affective spectrum, such as anxiety disorders, depression, or burnout (13–16). Therefore, the current study focuses on mental health professionals’ health concepts and beliefs related to depressive disorders and burnout. This choice is also substantiated by the ongoing controversy on whether depression and burnout constitute two distinct entities, or if they represent two different views of the same phenomenon (17): several studies found a strong nomological and psychometric overlap between both phenomena (18–20), while others came to the conclusion that the two constructs are distinct (21).

**FIGURE 1:**
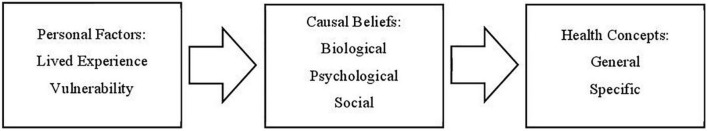
Theoretical model. Theorized relationships between study variables.

Building on the results of the EKB study and further literature (6, 22), the main hypothesis of the current paper is that lived experience of depression and perceived vulnerability to mental illness are related to mental health professionals’ causal beliefs concerning mental disorders, which again predict their general concept of mental health and their specific concepts of depression and burnout (see Figure 1). This hypothesis follows the basic research question whether and how depression and burnout are perceived as different phenomena depending on participants’ lived experiences of mental health problems. In the discussion, the practical implications of the found interrelations are discussed in relation to literature on the stigmatizing attitudes and behaviors toward persons with mental illness by both mental health professionals and the general public.

## 2. Materials and methods

### 2.1. Sampling

Following the qualitative research phase, an online survey was conducted among mental health professionals in the German federal states of Berlin and Brandenburg (12). The survey was completed by 218 professionals with direct patient contact from 18 psychiatric hospital departments (see Table 1).

**TABLE 1:** Sample characteristics.

		Female		Male		Diverse			
**Profession**	***N***	***n***	**%**	***n***	**%**	***n***	***%***	***M*_Age_**	***SD*_Age_**
Total	218	158	72.5	58	26.6	1	0.5	41.4	10.5
Social workers	15	13	86.7	1	6.7	1	6.7	44.0	9.8
Nurses	72	49	68.1	23	31.9	–	–	42.0	9.8
Psychologists	42	35	83.3	7	16.7	–	–	36.9	9.4
Physicians	56	33	58.9	22	39.3	–	–	40.6	10.4
Peer workers	3	3	100.0	–	-	–	–	41.7	11.1
Special therapists and miscellaneous	30	25	83.3	5	16.7	–	–	46.2	11.9

One participant did not indicate a gender; four participants did not indicate their age.

### 2.2. Measures

#### 2.2.1. Perceived vulnerability

To understand more on the perceived vulnerability to develop a mental illness, the participants were shown a modified version of the Self-Identification as Having a Mental Illness (SELF-I) scale (23). This instrument originally consists of five items and assesses the extent to which given symptoms are interpreted as indicators of a mental illness (e.g., “I am the type of person that could be prone to having a mental illness”). As one of the five items refers to current symptoms, it was omitted from the survey. The remaining four items were slightly reworded for the purpose of the current study (“mental crisis” instead of “mental illness”). The items were rated on a 5-point Likert scale (0 = *not at all*, 4 = *entirely*).

#### 2.2.2. Lived experience

Further, the participants were asked if they had ever experienced episodes of mental crisis including mental disorders, and if so, to assign their experience(s) to one or several DSM-based diagnostic categories (for details, see 12). For the purpose of the current study, self-assignment to the “depression” category was used to differentiate participants with lived experience of depression from those without such experiences. As the EKB study, for conceptual reasons, focused only on DSM-based diagnostic categories, lived experiences of burnout were not investigated.

#### 2.2.3. Causal beliefs

To assess the participants’ causal beliefs regarding mental illness in terms of the biopsychosocial model of health, four factors were presented based on previous studies (24, 25), namely “biological and genetic factors,” “current stress,” “traumatic childhood experiences,” and “societal conditions.” Participants were asked to rate the influence of each factor on the emergence of mental disorders on a 5-point Likert scale (0 = *not at all*, 4 = *entirely*).

#### 2.2.4. General health concept

The basic concept of mental health was gauged in terms of continuum beliefs. Participants were asked to rate their agreement to the following statement, modified from (6). “Basically we all sometimes experience mental crises, it is just a question how pronounced this state is.” The answers were given on a 5-point Likert scale (0 = *not at all*, 4 = *entirely*), with low agreement indicating a rather categorical concept of mental health.

#### 2.2.5. Specific concepts of depression and burnout

The participants’ specific concepts toward depression and burnout were assessed *via* eight statements derived from a qualitative preliminary study (see Table 2). In order to capture any similarities and dissimilarities between the two conditions, these statements were presented referring to both depression (e.g., “Depression is a result of too much work”) and burnout (e.g., “Burnout is a result of too much work”). Participants were asked to indicate their agreement to the 16 items on a 5-point Likert scale (0 = *not at all*, 4 = *entirely*).

**TABLE 2:** Concepts of depression and burnout.

		Depression		Burnout			
	**Depression/Burnout…**	***M***	***SD***	***M***	***SD***	***t (217*)**	***P***
1	… is the result of too much work	1.43	1.03	2.56	1.06	-14.0	<0.001
2	… is strongly influenced by genetic disposition	2.67	0.96	1.43	1.02	16.5	<0.001
3	… heals without long-term consequences	1.59	0.93	1.90	1.03	-4.5	<0.001
4	… affects all areas of life	3.50	0.73	3.21	0.85	5.0	<0.001
5	… affects people who are very committed	1,70	0.93	2.34	1.06	-8.8	<0.001
6	… is a condition that you have to deal with for the rest of your life	2.11	1.17	1.42	1.02	8.8	<0.001
7	… can distinguish a person positively	1.65	1.08	1.58	1.10	1.1	0.271
8	Someone with … is not well-adjusted to the societal conditions	1.17	1.00	1.22	1.00	-0.6	0.524

*N* = 218.

In addition, participants were asked to rate their agreement to the following comprehensive statement (entity distinction) which was also derived from the preliminary study: “Depression and burnout are two different conditions.” The answer was to be given on a 5-point Likert scale (0 = *not at all*, 4 = *entirely*).

### 2.3. Data analysis

All statistical analyses were conducted with STATA (26). The study variables were explored using descriptive statistics.

To examine the factor structure of the SELF-I scale, confirmatory and exploratory factor analyses (CFA and EFA, respectively) were conducted. Scale reliability was evaluated by testing the internal consistency using Cronbach’s alpha. The scale was constructed by calculating the mean, with high values indicating high self-assessed vulnerability to mental crises.

The two causal factor variables representing psychological causes (“current stress” and “traumatic childhood experiences”) were combined into one variable.

Comparisons between participants’ agreements to the parallel items regarding the concepts of depression and burnout, respectively, were drawn using paired *t*-tests. Further, the absolute differences between the respective parallel items were calculated and combined into a new variable by calculating the mean (depression-burnout differentiation score). This variable was used as a measure to quantify the conceptual discrimination between depression and burnout.

A correlation analysis was conducted to assess for age, gender, and educational level as potential covariates.

**FIGURE 2:**
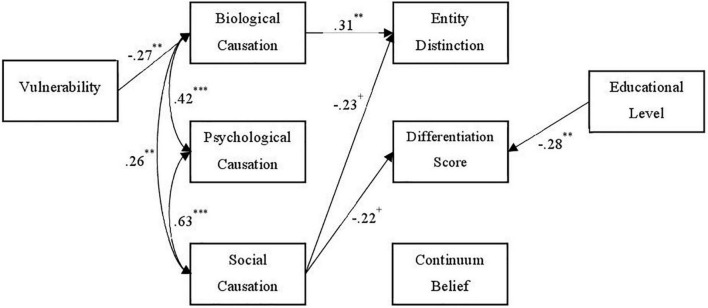
Path model (no depression group). N = 92. Standardized path coefficients. Nonsignificant paths are not displayed. Coefficients with *p* < 0.10 were included due to the small study sample. ^+^*p* < 0.10, ^**^p < 0.01, ^***^p < 0.001.

**FIGURE 3:**
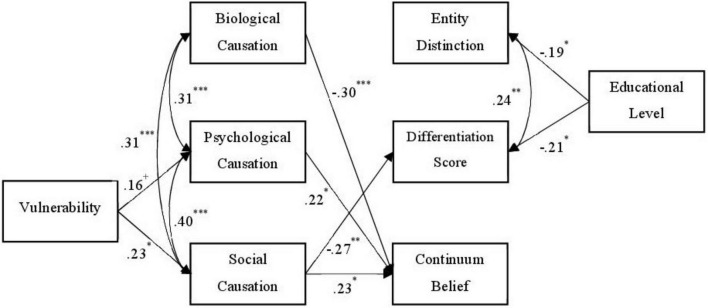
Path model (depression group). N = 126. Standardized path coefficients. Nonsignificant paths are not displayed. Coefficients with *p* < 0.10 were included due to the small study sample. ^+^*p* < 0.10, *p < 0.05, ^**^p < 0.01, ^***^p < 0.001.

To test the hypothesized relationships between the study variables, a path model was constructed with the participants’ perceived vulnerability as predictors, the causal beliefs as mediating variables, and variables representing participants’ general and specific health concepts as criteria (see Figures 2, 3). To compare participants with and without previous depression experience, a group analysis was conducted with lived depression experience as grouping variable. Multivariate normality, a prerequisite for the use of path models, was tested using Mardia’s normalized estimates of multivariate kurtosis and skewness (27), which revealed non-normality. Therefore, the path analysis was conducted using bootstrap calculations.

## 3. Results

Descriptive analyses of all items used in the current study revealed acceptable values.

### 3.1. Scales and variables

#### 3.1.1. Perceived vulnerability

Self-identification as having a mental illness is regarded a one-dimensional construct (23). A CFA with one latent factor showed poor model fit, χ^2^ = 31.95, df = 2, *p* < 0.001, CFI = 0.83, RMSEA = 0.26, 90% KI (0.19, 0.35), SRMR = 0.10. An EFA (principal axis factor analysis) was conducted on the four items. One of the extracted factors met Kaiser’s criterion (28). However, the scree plot was ambiguous, and a parallel analysis suggested two factors. Therefore, the four items were combined by calculating the mean. The internal consistency of the SELF-I scale was α = 0.68, just below the common threshold of 0.70 (29). Participants indicated an average vulnerability of *M* = 1.87 (*SD* = 0.74).

#### 3.1.2. Lived experience with depression

Of the 218 participants, 126 (57.8%) indicated previous episodes of depression. The depression group (*M*_Age_ = 41.7, *SD*_Age_ = 10.4, female = 77.0%, male = 23.0%) and the no-depression group (*M*_Age_ = 40.9, *SD*_Age_ = 10.6, female = 66.3%, male = 32.6%, diverse = 1.1%) showed similar characteristics in terms of age and gender.

#### 3.1.3. Causal beliefs

A correlation analysis of the four causal factor items revealed substantial positive relationships between all items (ranging from *r* = 0.28 to 0.57), indicating that the participants’ on average did not favor one of the causes above others. Average agreement to biogenetic and social factors as causes of mental illness was *M* = 3.06 (*SD* = 0.85) and *M* = 3.07 (*SD* = 0.86), respectively. The two items referring to psychological causes were combined into one “psychological” factor by calculating the mean, with an average agreement of *M* = 3.48 (*SD* = 0.64; Cohen’s α = 0.73).

#### 3.1.4. General health concept

On average, participants indicated a rather high agreement to a continuum of mental health and crisis, *M* = 3.23 (*SD* = 0.88).

#### 3.1.5. Specific concepts of depression and burnout

The participants significantly differentiated between depression and burnout in six of the eight parallel items (see Table 2). The combination of the absolute differences between the parallel items revealed an overall differentiation score of *M* = 0.85, *SD* = 0.40.

Participants agreement to entity distinction regarding depression and burnout on average amounted to *M* = 2.56 (*SD* = 1.35).

### 3.2. Correlation analysis

A correlation analysis (see Table 3) showed a significant relationship between participants’ gender and their previous depression experiences (*r* = –0.13, *p* = 0.049), indicating that the women in the current sample had more frequently experienced depressive episodes compared to male and diverse participants.

**TABLE 3:** Study variable correlations.

	Variable	1	2	3	4	5	6	7	8	9	10	11
1	Depression experience	1	0.39***	0.13	0.01	0.15*	0.07	0.00	-0.14*	0.04	-0.13*	0.01
2	SELF-I	–	1	0.09	-0.10	0.11	0.16*	-0.10	-0.12	-0.03	-0.11	-0.04
3	Continuum belief	–	-	1	-0.07	0.24***	0.25***	-0.01	0.08	-0.07	-0.06	0.02
4	Biogenetic causation	–	-	-	1	0.37***	0.28***	0.08	0.17*	0.01	-0.03	-0.13
5	Psycho-logical causation	–	-	-	-	1	0.52***	0.00	0.02	0.13	-0.10	-0.07
6	Social causation	–	-	-	-	-	1	-0.18*	-0.02	-0.02	-0.07	-0.07
7	Differentiation score	–	-	-	-	-	-	1	0.25***	0.09	0.00	-0.24***
8	Entity distinction	–	-	-	-	-	-	-	1	0.12	0.03	-0.19
9	Age^a^	–	-	-	-	-	-	-	-	1	-0.02	-0.26***
10	Sex^b,c^	–	-	-	-	-	-	-	-	-	1	0.01
11	Educational level^b,d^	-	-	-	-	-	-	-	-	-	-	1

*N* = 218. SELF-I, self identification as having a mental illness scale.

^a^*n* = 214.

^b^*n* = 217.

^c^0 = Female, 1 = Male, 2 = Diverse.

^d^1 = Up to 10 years of school, 2 = More than 10 years of school.

*p < 0.05, **p < 0.01, ***p < 0.001.

The correlation analysis also revealed significant negative correlations between educational level on the one hand and the differentiation score (*r* = –0.24, *p* < 0.001) and entity distinction item (*r* = –0.19, *p* = 0.005) on the other hand, meaning that participants with lower educational level tend to regard depression and burnout as different concepts.

Apart from these results, no further significant relationships of age, gender, and educational level with other study variables emerged.

Because of the significant correlations between both “depression-burnout-differentiation-variables” on the one hand and educational level on the other hand, the latter was added to the path model as a predictor of the former.

### 3.3. Path model

The overall fit of the path model was acceptable, χ^2^ = 13.358, df = 14, *p* = 0.499, CFI = 1.00, RMSEA = 0.00, 90% KI (0.00, 0.09).

The path model comparison between participants with and without previous depression experiences revealed quite different path patterns between the study variables (see Figures 2, 3, respectively).

In participants without lived depression experiences, SELF-I negatively predicted the belief in biological causation, which in turn positively predicted entity distinction. Both entity distinction and the differentiation score were negatively predicted by social causation, but slightly outside the conventional significance level of *p* = 0.05 (*p* = 0.066 and 0.069, respectively). Educational level significantly and negatively predicted the differentiation score.

In the participants with previous depression experiences, SELF-I positively predicted psychological and social causation. Agreement to the continuum statement was negatively predicted by the agreement biological causation, and positively by agreement to psychological and social causation. The differentiation score, but not entity distinction, was negatively predicted by the agreement to social causation. Educational level negatively predicted both depression-burnout-disparity-variables.

In neither group, the belief in psychological causation was significantly related to the depression-burnout-disparity-variables.

## 4. Discussion

Our study demonstrates that mental health professionals with lived depressive experience conceptualize depression and burnout, as well as the overlap between the two constructs, differently compared to colleagues without such experiences. In the subgroup of participants with lived experiences, a higher degree of perceived vulnerability to mental illness was associated with higher appraisal of societal causation of mental illness, which again was linked to higher continuum beliefs, and lower rates of discrimination between depression and burnout.

While the latter association was also found in the subgroup of participants without lived experience, among these participants, an additional, reverse mechanism seemed to be at work which increased the depression-burnout-discrimination. Namely, in this subgroup a higher degree of perceived vulnerability to develop a mental illness was associated with lower levels of agreement to biological causation of mental illness, which was linked to higher rates of discrimination between depression and burnout.

### 4.1. Concepts matter

Participants without own experience of depression and with a rather biogenetic understanding of mental illness were more likely to divide depression and burnout into two different categories (entity distinction), while participants with own lived experience and rather social causal beliefs tended to see the concepts more closely together (differentiation score).

The causal beliefs of participants with depression experience were not relevant to specifying between burnout and depression but shaped their general concept of mental health. This connection could be explained by a deeper reflection process in persons with lived experiences, linking questions of causes with thoughts on diagnostic conceptualization. Due to personal confrontation with a mental problem, they may have been forced to explore and reflect deeper upon such problems. These connections were not found in people who have not experienced depression, suggesting that causal beliefs in this subgroup may be less personal and more likely to be based on theoretical knowledge and observations, resulting in a rather pronounced distinction between depression and burnout.

The belief in a biogenic causation of mental illness has a separating effect on both the distinction between depression and burnout (specific health concept) and on the demarcation between mentally sick and healthy (general health concept), while the belief in a social causation has a unifying effect. The negative influence of biogenetic causal beliefs on continuum beliefs in the depression group seems surprising at first glance. Previous studies have already shown that a biogenetic causal belief can lead to greater stigmatization and othering (30, 31). Processes such as self-stigmatization and othering of one’s own person are well-known phenomena. The lower level of continuum beliefs could be an expression of this.

Thus, our study shows that both the general and specific health concepts of mental health professionals are influenced by causal beliefs. Whether and how strong this influence is depends on one’s own lived experiences.

### 4.2. Experiences matter

Further, our study demonstrates that the participants’ perceived vulnerability to develop a mental illness influences their causal beliefs, and therefore effects their general and specific health concepts (of depression and burnout) indirectly. While in the participants with lived depression experience higher perceived vulnerability was linked to higher beliefs in psychological and social causation of mental illness, no effect from vulnerability on biological causation beliefs was found. In participants without depression experiences, a different pattern was found, with higher vulnerability being associated with less beliefs in a biological causation of mental health but not being linked to the other two causation types. It seems that the perception of one’s vulnerability to mental health problems has different meanings for the participants, depending on whether they had previous experiences with depression or not.

More generally, the general concept of mental health and the specific conceptualization of depression and burnout were indirectly influenced by the perceived vulnerability to develop a mental health disorder *via* causal beliefs. People with own experiences of depression who considered themselves more vulnerable believed more strongly in psychosocial causation, which in turn reinforced the belief that everyone can be affected (continuum belief). People without personal experience who considered themselves less vulnerable tended to stronger believe in biological causation and therefore in turn that depression and burnout were two different phenomena.

### 4.3. Education matters

Participants’ level of education was significantly linked to the differentiation score between depression and burnout in both subgroups, and to entity distinction between the two constructs in the depression subgroup. Higher educational level (i.e., more than 10 years of school) was associated with less discrimination between depression and burnout, but not with continuum beliefs. Perhaps this can be explained by different levels of mental health literacy and/or different professional groups, both leading to different knowledge and understanding of the concepts of depression and burnout. In any case, this interesting finding should be further investigated in future research.

### 4.4. Practical implications

Our results have several practical implications. The health concepts of professionals have an impact on clinical practice. Previous studies have shown that higher continuum beliefs are associated with more pro-social emotional reactions toward and lower desire for distance from individuals with mental illness in the general public (6, 32). Thus, the perception of categorical differences between individuals with and without mental health problems is a substantial step of stigmatizing processes, leading individuals who identify themselves as “mentally healthy” to distance themselves from those labelled as “not mentally healthy.”

In relation to the distinction between depression and burnout, the belief that depression is a mental disorder or illness related to stigmatizing attitudes is still widespread (33). In contrast, burnout is mostly regarded as a non-medical and work-related phenomenon associated with relatively low stigmatization (34). In an interview study, Bahlmann et al. (35) demonstrated that the use of the label “depression” instead of “burnout” for the same case vignette was associated with significantly higher desire for social distance, which is a central element of the stigma process (36). In an online survey, Bianchi et al. (37) found significantly stronger stigmatizing attitudes toward depression compared to burnout. Due to the higher stigmatization of depression compared to burnout, an attitude which includes a more pronounced distinction between the two concepts may be indicative of a rather stigmatizing attitude toward mental health problems.

Considering this background, it is interesting that our results indicate that the causal beliefs of participants who have not experienced depression are important to a discrimination between burnout and depression, while those who are affected relate their understanding of causes more to a general health concept than to that specific discrimination.

Further, as shown in the introduction, the mental health professionals’ beliefs may also affect the service users’ beliefs (1). Accordingly, previous studies showed that the clinicians’ beliefs or concepts about the nature of illnesses plays an important role for the users’ health-related behavior (38). This makes it even more important to understand the practitioners’ beliefs and how they are formed. Other studies demonstrate that mental health professionals’ own causal beliefs may also affect their attitudes toward individuals with mental health problems (2). Unbalanced and dominating beliefs in a biological or biogenetical causation of mental disorders, i.e., genetic or physiological factors like heritability, chemical changes, or brain abnormalities, may have detrimental consequences. Previous studies indicate that a biological conceptualization of mental illness, while it can diminish blame, was associated with increased negative stereotypes and stigma of persons with mental illness in the public and among mental health staff (30, 31, 39–41).

Therefore, understanding professionals’ causal beliefs has clinical relevance. Our results show that the belief in a biological causation is associated with dichotomous concepts while a social understanding of causes rather unites the concepts. Biological factors are apparently understood as a predisposition which fundamentally differentiates people, whereas society shapes people rather collectively. The resulting tension between these causations can be resolved by considering that humans are a bio-psycho-social beings, with biological aspects influencing and being influenced by social and psychological aspects.

In an experimental study, participants with own experiences of psychiatric treatment showed significantly less negative attitudes toward psychiatric service users compared to participants without such experience (42). Further, higher self-perceptions of having a mental illness was associated with less stigmatizing attitudes (43) or intentions to stigmatize (44).

Among our participants, perceived vulnerability was related to reduced biological understanding of causes and, indirectly, with stronger differentiation between burnout and depression in people without depression experience. In people who have experienced depression, perceived vulnerability was associated with a higher belief in psychosocial causes and, indirectly, with stronger continuum beliefs.

In light of these findings, the openness to one’s own vulnerability can be considered as an important factor influencing mental health professionals’ attitudes toward their patients in terms of reduced need for social distance and stigmatization.

### 4.5. Limitations

Due to the restricted number of participants, the current research is exploratory in nature, restricting the generalizability of the reached conclusions. For the same reason, participants’ professional groups could not be included in the statistical analyses, limiting the gain of knowledge. Also, participants’ lived experiences with burnout were not assessed, as burnout does not constitute a diagnostic category in the DSM. An additional limitation of the current study is its cross-sectional design, prohibiting conclusions concerning causal relationships between variables.

## 5. Conclusion

Aligning with our findings and those cited in the introduction as well as the discussion above, it can be assumed that mental health professionals’ lived experiences influence their actions toward the patients, how they conceive of their mental health problems, what kind of remedies are imagined upon and what treatment options are offered or taken.

Thus, the mental health professionals are not “external” to the clinical situation. Our study demonstrates that not only their causal beliefs or health concepts but, even more private, also their own crisis experiences and perceived vulnerability influence the evaluation of the patients’ situations. This makes the clinical encounter a personal one: instead of being “neutral” or “objective,” the “clinical gaze” (45) seems to be strongly shaped by the mental health professionals’ own attitudes and, even more, personal experiences. Mental health professionals are not simply distanced “measuring instruments” but strongly involved counterparts in clinical relationships.

## Data availability statement

The raw data supporting the conclusions of this article will be made available by the authors, without undue reservation.

## Ethics statement

Ethical review and approval was not required for the study on human participants in accordance with the local legislation and institutional requirements. The patients/participants provided their written informed consent to participate in this study.

## Author contributions

All authors listed have made a substantial, direct, and intellectual contribution to the work, and approved it for publication.
